# Automated psychological therapy using immersive virtual reality for treatment of fear of heights: a single-blind, parallel-group, randomised controlled trial

**DOI:** 10.1016/S2215-0366(18)30226-8

**Published:** 2018-08

**Authors:** Daniel Freeman, Polly Haselton, Jason Freeman, Bernhard Spanlang, Sameer Kishore, Emily Albery, Megan Denne, Poppy Brown, Mel Slater, Alecia Nickless

**Affiliations:** a Department of Psychiatry, University of Oxford, Oxford, UK; b Nuffield Department of Primary Care Health Sciences, University of Oxford, Oxford, UK; c Oxford VR, Oxford, UK; d Oxford Health NHS Foundation Trust, Oxford, UK; e Virtual Bodyworks, Barcelona, Spain; f Department of Clinical Psychology and Psychobiology, Faculty of Psychology, University of Barcelona, Barcelona, Spain

## Abstract

**Background:**

Engaging, interactive, and automated virtual reality (VR) treatments might help solve the unmet needs of individuals with mental health disorders. We tested the efficacy of an automated cognitive intervention for fear of heights guided by an avatar virtual coach (animated using motion and voice capture of an actor) in VR and delivered with the latest consumer equipment.

**Methods:**

We did a randomised trial of automated VR versus usual care. We recruited adults aged older than 18 years with a fear of heights by radio advertisements in Oxfordshire, UK. We diagnosed fear of heights if participants scored more than 29 on the Heights Interpretation Questionnaire (HIQ). We randomly allocated participants by computer in a 1:1 ratio to either automated VR delivered in roughly six 30-min sessions administered about two to three times a week over a 2-week period (intervention group) or to usual care (control group). Randomisation was stratified by severity of fear of heights. The research team, who were unaware of the random allocation, administered three fear-of-height assessments, at baseline (0 weeks), at the end of treatment (2 weeks), and at follow-up (4 weeks). The primary outcome measure was HIQ score (range 16–80, with higher scores indicating greater severity). This trial is registered with the ISRCTN registry, number ISRCTN11898283.

**Findings:**

Between Nov 25, 2017, and Feb 27, 2018, 100 individuals were enrolled and underwent randomisation, of whom 49 were assigned to the VR treatment group and 51 to the control group. All participants completed the 4-week follow-up. The mean total treatment time in VR was 124·43 min (SD 34·23). Compared with participants in the control group, the VR treatment reduced fear of heights at the end of treatment (mean change score −24·5 [SD 13·1] in the VR group *vs* −1·2 [7·3] in the control group; adjusted difference −24·0, 95% CI −27·7 to −20·3; Cohen's d=2·0; p<0·0001). The benefit was maintained at follow-up (mean change score −25·1 [SD 13·9] in the VR group *vs* −1·5 [7·8] in the control group; adjusted difference −24·3, 95% CI −27·9 to −20·6; Cohen's d=2·0; p<0·0001). The number needed to treat to at least halve the fear of heights was 1·3. No adverse events were reported.

**Interpretation:**

Psychological therapy delivered automatically by a VR coach can produce large clinical benefits. Evidence-based VR treatments have the potential to greatly increase treatment provision for mental health disorders.

**Funding:**

Oxford VR, and the National Institute of Health Research Oxford Health Biomedical Research Centre.

## Introduction

Mental health disorders are very common and encompass great personal and societal costs, but far too few people receive the best treatments. For example, in the UK, one adult in six meets criteria for a common mental health disorder but most of these individuals do not receive treatment.^1^ People who do receive treatment are more likely to be given psychotropic medication than a psychological intervention. Yet, for many common mental health conditions, particularly anxiety disorders, evidence-based psychological treatments are both the best treatment option^2^ and the preference of patients.^3^ However, increasing the provision of psychological treatment is difficult. Therapists need to be trained and then adhere competently to evidence-based treatment protocols. Moreover, the best psychological treatments are not simply so-called talking therapies but take the form of direct active learning and coaching in real-world situations, whereas most therapists typically have little time for sessions outside the clinic. This situation results in high service pressures and unmet needs of patients.

Immersive virtual reality (VR) has the potential to substantially increase access to the best psychological interventions. First, treatments can be automated and provided in VR, so a therapist does not need to be present. Automated treatment delivered using VR consumer hardware could become a low-cost way of providing effective interventions at scale. Second, VR can deliver the most powerful element of direct therapeutic intervention—ie, direct coaching in everyday situations that trouble patients. This element is all too frequently missing in clinics. By putting on a headset, patients can be taken immediately into various situations, graded in difficulty, that they find cause psychological distress. Third, in VR, patients are willing to go into situations that trouble them and try alternative ways of responding, because they know it is a simulation. However, the learning transfers to the real world.^4^ Therapeutic intervention is therefore faster. Moreover, the engaging and entertaining nature of VR could result in higher treatment uptake than with conventional treatment. VR has been used successfully over the past 25 years for assessment, understanding, and treatment of mental health disorders.^5^ The increased accessibility and affordability of VR mean that this technique is now ready to move from specialist laboratories into clinics.

**Research in context**

**Evidence before this study**

At the beginning of 2017, we searched PubMed with no restrictions on date for all published work in the English language on use of virtual reality (VR) to assess, understand, and treat mental health problems. The general search terms were (“virtual reality” OR “immersive virtual reality”) AND (“assessment”, “treatment”, “research”, “study”, “experiment”, OR “understanding”) AND ([disorder-specific terms]). Our search retrieved 285 empirical papers. Over the past 25 years, VR has been used to aid therapist-delivered psychological interventions, principally exposure therapy for anxiety disorders. Meta-analyses indicate that treatment effect sizes using VR for anxiety disorders, including fear of heights, are large (Cohen's d=1·1). However, VR-assisted therapy has only been delivered in a few places, because of the specialist equipment that is needed and the reliance on a skilled therapist to provide the psychological expertise. We found no reports of automated VR being used to treat mental health problems. We updated our search of PubMed on May 14, 2018, with no restrictions by language, with the terms (“VR” OR “virtual reality”) AND (“treatment”) AND (“mental health”) AND (“automated”, “self-guided”, OR “automatic”). We did not retrieve any further research.

**Added value of this study**

Our study comprised 100 individuals who self-referred for therapy. We showed that psychological treatment for fear of heights can be automated using immersive VR. Automation was delivered via a virtual coach, rather than a therapist, which guided participants through a series of graded exercises beside virtual heights that could facilitate cognitive change. Participants interacted with the virtual coach to tailor the treatment. The clinical effectiveness in our study was at least as good as face-to-face therapy.

**Implications of all the available evidence**

Automated treatment delivered using VR consumer hardware could become a low-cost way of providing effective interventions at scale. Clinical testing is needed to ascertain whether automation of psychological treatment works for other mental disorders.

Previous research using VR for treatment of mental health disorders has relied on having a therapist present, typically to deliver exposure therapy. Effect sizes for VR-assisted therapy are usually large (Cohen's d=1·1).^6^ The first application of VR to mental health difficulties was for treating fear of heights,^7^ which is the most common phobia. One in five people report having had a strong unreasonable fear of heights during their lifetime, and one in 20 people reach diagnostic criteria for acrophobia.^8^ One of the largest trials for this disorder randomly allocated 33 people with a fear of heights to three 1-h sessions of either exposure in vivo or VR exposure—in both cases delivered by a therapist.^9^ Both treatments were equally effective and the benefits remained 6 months later. The therapeutic effects of VR exposure for fear of heights persist for at least a year.^10^

We aimed to automate the provision of a psychological intervention for fear of heights by programming a virtual coach to act as the therapist in VR. Our therapeutic approach is cognitive, focusing on the evaluation of threat predictions while dropping defensive behaviours, to develop memories of safety that counteract fear associations. The treatment is delivered using inexpensive consumer VR equipment. We postulated that, compared with usual care (in effect, no treatment), a large treatment benefit (reduction in fear of heights) would be seen with the VR intervention immediately after treatment (primary hypothesis) and that this benefit would persist after treatment had been completed (secondary hypothesis).

## Methods

### Study design and participants

We did a single-blind, parallel group, randomised controlled, superiority trial comparing automated VR with usual care. The control condition was usual care so that we did not interfere with any other type of help that the individual might have been receiving. In effect, this was equivalent to receiving no treatment for fear of heights. At the end of the trial, control participants were offered the opportunity to have the VR treatment.

We recruited people with a fear of heights by advertisements on local radio (Oxfordshire, UK). Inclusion criteria were that a participant had to be older than 18 years and have a fear of heights corresponding to a Heights Interpretation Questionnaire (HIQ)^11^ score of more than 29. We used the same cutoff as was used in the treatment trial by Arroll and colleagues,^12^ which indicates at least a moderate fear of heights. We excluded people if they were currently receiving a psychological treatment for fear of heights, had photosensitive epilepsy, or had either no stereoscopic vision or balance problems.

The study received ethics approval from the University of Oxford Medical Sciences Inter-Divisional ethics committee. All participants gave written informed consent for trial participation. No changes to methods were made after the trial began.

### Randomisation and masking

Randomisation was done by an automated online system developed by the University of Oxford Primary Care Clinical Trials Unit. Participants were randomly allocated in a 1:1 ratio, stratified by fear of height severity (moderate [HIQ score 30–55] or severe [HIQ score 56–80]) and using randomised permuted block sizes of four and eight. The research assistant who gathered follow-up data was unaware of the random allocation, and masking was not broken.

### Procedures

After a potential participant contacted the study team, we asked them to complete the HIQ online. If they scored above the threshold (HIQ score >29), we checked inclusion and exclusion criteria over the telephone. Participants needed to score above the HIQ threshold again when they attended for the baseline assessment (and were excluded before randomisation if they did not). All assessments were done at the study centre (Oxford VR, Oxford, UK). At baseline, we obtained basic demographic data and assessed participants with the DSM-5^13^ to see if they met criteria for acrophobia. We also asked participants if they were receiving any treatment for fear of heights.

The VR intervention consisted of a software application called *Now I Can Do Heights*, which was intended by Oxford VR to help users with acrophobia overcome their fear of heights. The application is intended for use by adults older than 18 years and was designed to be used without a supporting therapist, although it can be used alongside a therapist or in a clinical setting if needed. The application is a CE-marked class I active medical device (device code Z301 [standalone software]), in conformity with the essential requirements and provisions of EC directive 93/42/EEC (medical devices). The software was developed using Unity3D (version 5.6.0f3 [64-bit]; Unity Technologies, San Francisco, CA, USA) and delivered using a gaming personal computer (Chillblast Fusion Strix Gaming PC, Intel Core i7-7700K processor, 16 GB DDR4 3000 MHz memory, ASUS GeForce GTX 1080 8GB graphics card, 500 GB M.2 solid state drive/3 TB hard disc drive; Chillblast, Poole, UK) and the HTC Vive (HTC Corporation, New Taipei City, Taiwan)—a consumer VR head-mounted display that has associated hand controllers and headset tracking. Participants also wore headphones with a microphone (Creative Labs Sound Blaster Tactic3d Rage Wireless; Creative Labs, Jurong East, Singapore).

The treatment was designed to be administered in roughly six 30-min VR sessions over a period of 2 weeks. We allowed a degree of flexibility in treatment length because individuals differ in the speed with which they progress. Participants underwent the treatment in the office of Oxford VR, with a graduate psychologist in the room to help the participant put on the headset and for basic safety reasons. Programme sessions ran automatically. At the beginning of every session, the participant's virtual body was calibrated by the software system reading data from head and hand VR trackers. Participants who were assigned the VR intervention undertook the treatment while standing up, and they could walk in the virtual environment.

At the beginning of the first session, the participant had an initial assessment with the virtual coach in a virtual office. The virtual coach was animated using motion capture from an actor and voiced by the same person. The coach gave the participant basic information about fear of heights and its treatment from a cognitive perspective (eg, “The reason we're afraid of heights is because we think something bad is going to happen. And that makes us feel anxious. Then we end up avoiding heights because they feel so scary. But I'll show you how to look at those thoughts in a new way.”), then asked the participant a series of questions about their key fear about heights (eg, fear of falling, fear of the building collapsing, fear of throwing oneself off) and obtained ratings of belief conviction (rated on a scale from 0 [“I don't believe it will happen”] to 10 [“I'm absolutely certain it will happen”]). The basic mechanism of treatment was for individuals to find out how accurate their fears were. Hence, throughout the programme, participants were encouraged by the virtual coach to find out how safe they were and to put their expectations to the test (eg, “Remember: we're exploring here. We're testing out our expectations. We're discovering what happens when we venture into a situation we'd normally try to avoid.”). The virtual coach also explained how this learning depended on dropping safety-seeking behaviours (eg, “Many people try to deal with their fear of heights by using defences. They put up barriers between themselves and what they fear. The most obvious one is simply avoiding heights. But there are lots of subtler defences: closing your eyes when you're up high or not looking down, repeating a comforting phrase to yourself, taking off your shoes, holding on to something. We need to lower these defences. They can make us feel better in the short term. But they prevent us truly engaging with the situations that make us anxious—and stop us learning just how much we can achieve without them.”). The treatment was not designed as exposure therapy (ie, participants were not asked to remain in situations until anxiety reduced) but as repeated behavioural experiment tests (ie, to learn that they were safer than they had thought). Throughout the sessions, participants' responses to questions from the virtual coach were given either by means of voice recognition technology (via a microphone attached to the headphones) or by using a virtual watch. Belief ratings were repeated within VR at the end of every treatment session.

After the initial assessment stage (during the first session), the treatment began while still within the first session. The virtual coach took the participant to the atrium of a large ten-storey office complex. The environment featured many height cues (eg, balls in the air, people moving about). The participant then chose on which of the first five floors to begin the activities (ie, participants could not access higher than the fifth floor until later sessions). Guided throughout by the virtual coach, tasks were undertaken on every floor to enable the participant to find out whether his or her fears were accurate. Tasks were randomised across all the remaining floors for every participant, but were weighted towards the easiest tasks first (eg, a barrier gradually lowering) whereas harder tasks came later (eg, going out on a platform from the floor into the atrium space). Many tasks were designed to be engaging for participants (eg, rescuing a cat from a tree, playing a xylophone near the edge of the floor, throwing balls over the edge of the floor). The virtual coach described the tasks to the participant, provided empathic encouragement, repeated key learning points, and sought feedback on whether the participant felt safer than before. The participant could decide to repeat tasks or move up to the next floor. At the end of the session, the participant was brought back down to the atrium ground level and the coach would ask for belief ratings for the height threat and encourage the participant to try real heights between sessions. The sessions were saved so that the participant could begin the next session where they had left off. Pictures from the VR treatment can be seen in the appendix.

### Outcomes

The primary outcome measure was the HIQ,^11^ which is a 16-item self-report questionnaire. The scale strongly predicts distress, anxiety, and avoidance of real heights and has high internal consistency and convergent validity with other fear of heights measures.^11^ We asked participants to rate anxious fears (eg, that they will fall, faint, or hurt themselves) when imagining two different height situations (ie, being on a ladder against a two-storey house and on the balcony of a 15th floor building). Scores can range from 16 to 80, with higher scores indicating a greater severity of fear of heights. Internal reliability of the scale at baseline was very high (Cronbach's α=0·91).

Secondary outcome measures were the acrophobia questionnaire (AQ)^14^ and the Improving Access to Psychological Therapies (IAPT) phobia scale–avoidance.^15^ The AQ is a 40-item self-report questionnaire. For 20 different height situations (eg, diving off the low board at a swimming pool, riding a Ferris wheel, walking on a footbridge over a motorway), we asked participants to rate their level of anxiety (on a scale from 0 to 6) and level of avoidance (scale from 0 to 2). We measured the AQ total score and two AQ subscale scores (anxiety and avoidance). Anxiety scores can range between 0 and 120 and avoidance scores between 0 and 40, with total AQ scores ranging between 0 and 160.^14^ Higher scores indicate a greater severity of fear of heights. The internal reliability of the scale at baseline was very high (Cronbach's α=0·90).

The IAPT phobia scale–avoidance is a single-item scale taken from the routine outcome measures administered by the UK National Health Service's IAPT programme, which treats common emotional disorders.^15^ We asked participants to rate their avoidance of heights on a scale from 0 (would not avoid it) to 8 (always avoid it). Higher scores indicated greater avoidance.

We also recorded the occurrence of any known serious adverse events in participants, which we defined as death, suicide attempts, serious violent incidents, admissions to psychiatric hospital, and formal complaints about the intervention. We used the Simulator Sickness Questionnaire (SSQ)^16^ to assess levels of discomfort provoked by a VR session. The SSQ is the most commonly used assessment of VR simulator sickness, but it was not developed in the context of the treatment of anxiety, and the 16 items in the scale overlap completely with symptoms of anxiety—eg, increased salivation, sweating, nausea, vertigo, burping, headache, blurred vision, and feeling dizzy. We administered the SSQ four times: before and after the first and last VR treatment sessions. Participants rated the items for how much they were experienced at that time, on a 4-point scale (from 0 [none] to 3 [very strong]). We used a simple raw total score of all items,^17^, ^18^ because we wanted to see absolute levels and potential increases in discomfort. The total score, therefore, can range between 0 and 48, with higher scores indicating greater discomfort.

### Statistical analysis

A full statistical analysis plan was agreed before the trial analysis (appendix). We did analyses with Stata version 15.1. Analyses were done by intention to treat at the end of the trial and were validated by a second statistician.

Because effect sizes for VR-assisted therapy for anxiety disorders are typically large (Cohen's d=1·1),^6^ our target sample size was 100 individuals, which would enable the trial to detect a standardised treatment effect of 0·65 (medium) with 90% power, and of 0·57 (medium) with 80% power, at a significance level of 0·05. These treatment effects amount to differences between treatments on the HIQ of 7·6 and 6·6, respectively, based on an SD of 11·7 (taken from the mean of the baseline SDs of the two groups reported by Arroll and colleagues).^12^

We analysed continuous outcomes using a linear mixed effects model, which included a random effect for participant to account for repeated measures at 2 weeks and 4 weeks. We included as fixed effects treatment group, assessment timepoint (as a categorical variable), baseline score for the outcome scale, and the interaction between treatment group and assessment timepoint (to allow estimation of a treatment effect at each of the two timepoints). In the secondary outcome analyses, we also included baseline HIQ scores as a fixed effect because stratification was based on this variable. We did not need techniques for missing data. We present results as mean differences in scores between treatment groups, with 95% CIs and associated two-sided p values. We calculated effect sizes using Cohen's d, dividing the treatment effect by the shared SD at baseline. We also ascertained the number needed to treat (NNT) to reduce the fear of heights by at least 25%, 50%, and 75%, and to below the study entry criterion.

This trial is registered with the ISRCTN registry, number ISRCTN11898283.

### Role of the funding source

Oxford VR own the automated treatment and helped design the trial. The National Institute for Health Research Oxford Health Biomedical Research Centre funded the randomisation programme and statistical analysis. Some of the authors are employed by the funders and contributed to the report. The decision to submit the trial results for publication was agreed during trial registration, before the trial began. The corresponding author had full access to all data in the study and had final responsibility for the decision to submit for publication.

## Results

**Figure 1:**
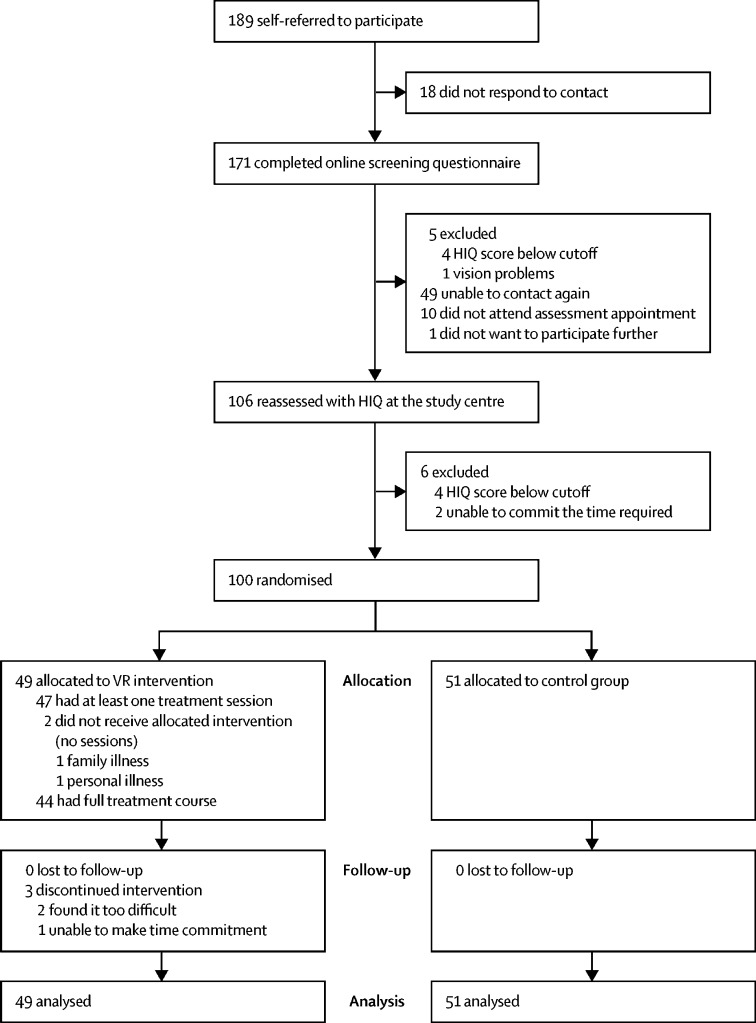
Trial profile HIQ=Heights Interpretation Questionnaire. VR=virtual reality.

Between October, 2017, and February, 2018, 189 people self-referred to the study, of whom 89 were excluded after screening and assessment (figure 1). Thus, 100 participants underwent randomisation, which began on Nov 25, 2017, and ended on Feb 27, 2018. 49 participants were allocated the VR intervention and 51 were assigned to the control group. Final data were gathered on April 6, 2018.

Participants had been experiencing a fear of heights for a mean of 30 years (SD 14·47). No participant was receiving any other form of help for their fear of heights. 90 participants met diagnostic criteria for acrophobia. Treatment groups were balanced across the variables (table 1), except for gender, with a higher proportion of women assigned to the control group. Scores on the HIQ were associated positively with scores on the AQ (*r*=0·68; p<0·0001) and with scores on the IAPT phobia scale (*r*=0·54; p<0·0001).

**Table 1:** Baseline characteristics

		**VR treatment group (n=49)**	**Control group (n=51)**
Age (years)		45 (30–53)	46 (38–53)
Men		29	19
Women		20	32
Ethnic origin			
	White	47	45
	Black African	0	1
	Black Caribbean	1	0
	Other	1	5
Employment status			
	Full-time employed	30	33
	Part-time employed	7	8
	Unemployed	2	3
	Retired	6	6
	Student	4	1
Duration of fear of heights (years)		32·0 (13·8)	28·4 (15·0)
Diagnosis of acrophobia		42	48

Data are number of participants, median (IQR), or mean (SD).

Uptake of the VR treatment was very high. 47 (96%) of 49 people attended at least one VR session. The mean number of treatment sessions attended by these 47 participants was 4·66 (SD 1·27). The mean length of a treatment session was 26·8 min (SD 2·7). The mean total VR treatment time for these individuals was 124·43 min (SD 34·23). 44 (90%) people had a full course of the VR intervention (ie, they completed all the treatment exercises), which comprised three (n=4), four (n=18), five (n=8), six (n=12), seven (n=1), or eight (n=1) treatment sessions. Three (6%) people did not complete the intervention, with two people finding the VR sessions too difficult (attending three and four sessions) and one person unable to attend further appointment times (attending one session).

**Figure 2:**
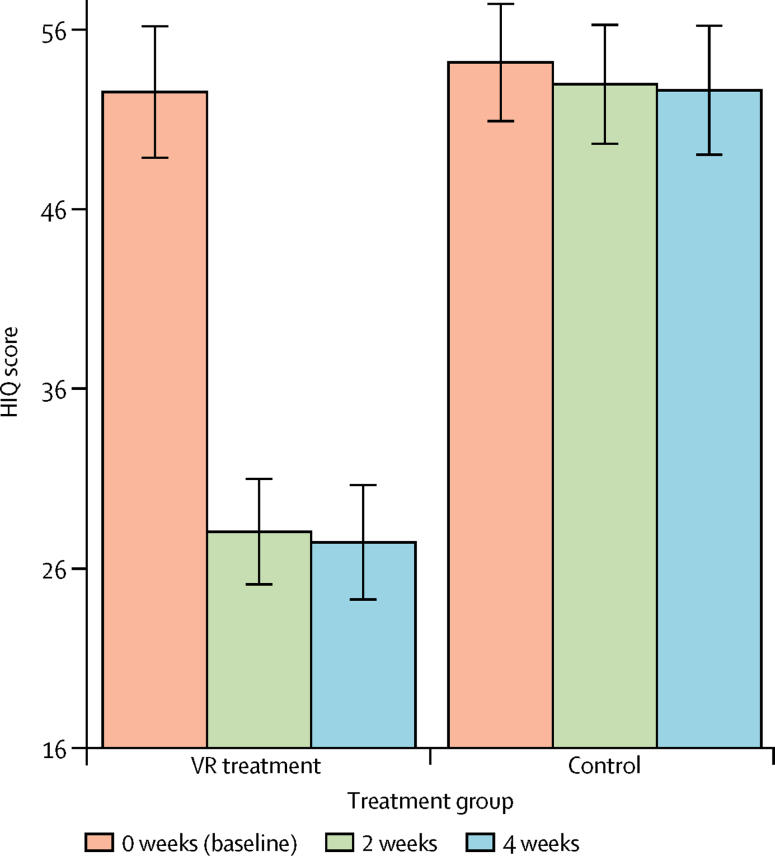
Scores on the HIQ at every timepoint for each randomised group The minimum score on the HIQ is 16. Bars represent the mean, error bars the 95% CI. HIQ=Heights Interpretation Questionnaire. VR=virtual reality.

All participants were followed up at all timepoints (baseline, 2 weeks, and 4 weeks) and no outcome data were missing. Table 2 summarises the mean scores of the primary and secondary outcome measures at every assessment point for each treatment group and the adjusted differences between groups. Participants allocated to the VR treatment group had very large reductions in scores on the three fear-of-heights assessments from baseline to 4 weeks, whereas scores for the control group remained stable (figure 2).

**Table 2:** Outcome measure scores at every timepoint and differences between groups

	**VR treatment group (n=49)**	**Control group (n=51)**	Adjusted group difference (95% CI)*	**Effect size (Cohen's d)**	**p value**
**HIQ total**					
0 weeks	52·5 (12·7)	54·2 (11·6)	..	..	..
2 weeks	28·1 (10·2)	53·0 (11·8)	−24·0 (−27·7 to −20·3)	2·0	<0·0001
4 weeks	27·5 (11·1)	52·6 (12·8)	−24·3 (−27·9 to −20·6)	2·0	<0·0001
**AQ total**					
0 weeks	71·9 (26·6)	70·3 (22·6)	..	..	..
2 weeks	29·1 (19·8)	73·9 (23·6)	−45·2 (−52·1 to −38·2)	1·8	<0·0001
4 weeks	25·1 (19·3)	69·9 (22·1)	−45·1 (−52·1 to −38·2)	1·8	<0·0001
**AQ anxiety subscale**					
0 weeks	59·0 (20·8)	58·0 (18·8)	..	..	..
2 weeks	24·0 (16·3)	61·1 (19·5)	−37·3 (−43·0 to −31·6)	1·9	<0·0001
4 weeks	20·4 (15·7)	57·3 (18·1)	−37·2 (−42·9 to −31·5)	1·9	<0·0001
**AQ avoidance subscale**					
0 weeks	12·9 (6·6)	12·3 (5·1)	..	..	..
2 weeks	5·1 (4·2)	12·9 (5·5)	−8·0 (−9·6 to −6·4)	1·4	<0·0001
4 weeks	4·7 (4·4)	12·6 (5·7)	−8·0 (−9·6 to −6·4)	1·4	<0·0001
**IAPT phobia scale**					
0 weeks	4·6 (1·9)	4·5 (2·0)	..	..	..
2 weeks	2·1 (1·6)	4·5 (1·6)	−2·3 (−2·9 to −1·8)	1·2	<0·0001
4 weeks	1·7 (1·5)	4·6 (1·7)	−2·9 (−3·4 to −2·3)	1·5	<0·0001

Data are mean (SD), unless otherwise indicated. AQ=acrophobia questionnaire. HIQ=Heights Interpretation Questionnaire. IAPT=Improving Access to Psychological Therapies. VR=virtual reality.

* Adjusted for treatment group, assessment timepoint, baseline score for the outcome scale, and the interaction between treatment group and assessment timepoint (HIQ, AQ, and IAPT); further adjustment was made for baseline HIQ score (AQ and IAPT). The difference was assessed by linear mixed effects models.

For the primary outcome analysis, the mean change in the HIQ total score at 2 weeks was −24·5 (SD 13·1) in participants allocated the VR intervention and −1·2 (7·3) in the control group. The adjusted difference in the treatment effect was −24·0 (95% CI −27·7 to −20·3; d=2·0; p<0·0001), in favour of the VR intervention (table 2). The mean change in the HIQ total score at 4 weeks was −25·1 (SD 13·9) in the VR treatment group and −1·5 (7·8) in the control group (adjusted difference in treatment effect −24·3, 95% CI −27·9 to −20·6; d=2·0; p<0·0001; table 2). 49 (100%) participants in the VR group showed a reduction in fear of heights on the HIQ, with the mean reduction being 68·0% (SD 26·6). By the follow-up timepoint, 34 (69%) of 49 people in the VR treatment group fell below the trial's fear of heights entry criterion on the HIQ, compared with none of 51 people in the control group (risk difference 0·61, 95% CI 0·48–0·75; NNT 1·6). In the VR group, 25 (51%) participants showed a reduction of 75% or greater (risk difference 0·45, 95% CI 0·31–0·59; NNT 2·2), 38 (78%) had a reduction of 50% or greater (0·78, 0·66–0·89; NNT 1·3), and 44 (90%) had a reduction of 25% or greater (0·86, 0·76–0·96; NNT 1·2). No participants in the control group showed a reduction of 75% or greater or 50% or greater, and nine (18%) had a reduction of 25% or greater. The mean reduction in the fear-of-heights score on the HIQ in the control group was 3·3% (SD 23·0). Outcomes were consistent with participant-described benefits of treatment (panel).

Panel **Participants' comments about VR treatment**

“What I'm noticing is that in day-to-day life I'm much less averse to edges, and steps, and heights, and I'm noticing in myself that when I'm doing the VR and outside I'm able to say ‘Hello’ to the edge instead of bracing against it and backing up. When I'm doing the VR I'm, as best as I'm able to, being open and curious around me as much as I can and noticing how the anxiety feels in my body, and then noticing that it goes really quickly now. So, when I've always got anxious about an edge I could feel the adrenaline in my legs, that fight/flight thing; that's not happening as much now. I'm still getting a bit of a reaction to it, both in VR and outside as well, but it's much more brief, and I can then feel my thighs soften up as I'm not bracing up against that edge. I feel as if I'm making enormous progress, and feel very happy with what I've gained.”

“Everything I thought it was going to be, it wasn't. I anticipated it was just going to be like a game, it was going to be something that wasn't going to arouse my senses. I found myself even after the third floor, fourth floor, going up, feeling nervous, anxious about what's about to happen next. It definitely pushed the limits in terms of what I thought I would be able to achieve, and then got me to go past that. Now that it's done, after my fourth session, I have to say I feel better for it. I've already been experimenting in the weeks to see what it would be like in a real-life environment. And what I would like to say is that it's absolutely brilliant, honestly, I do think it's made a huge difference. I do think my nervousness about heights is definitely a lot better.”

“I've just finished my sessions, I did four in total. Last week, after my third session, I went up to the Westgate [a shopping centre]; the difference in my mental capacity to deal with heights was amazing. Previously I wouldn't go anywhere near the edges, I was almost hanging right off, looking vertically down. The sessions I've had here have given me a lot to think about, and certainly with regards to my fear of heights it feels like it's helped a lot. So, very worthwhile doing.”

“I'm 60 years old and I've had a fear of heights, an extreme fear of heights, all my life. I came to the centre, I've had three sessions of VR and I've already surpassed everything that I imagined I could. I didn't actually think I'd be able to get to what is known as level 2 but I've achieved that. I am absolutely confident that I will be going to several other levels over the next week or two. For me, whilst it's not easy and I can't say that my fear of heights has gone, it has certainly improved and my confidence has improved so, so far so good!”

VR=virtual reality.

We did preplanned subgroup analyses by gender (appendix). Gender had an independent effect on several outcomes but no significant interaction terms were noted in the models between gender and treatment group (ie, gender did not moderate treatment effects). In both treatment groups, men seemed to do slightly better than women. In response to the imbalance observed in gender across the two treatment groups, we also did sensitivity tests adjusting the outcome analyses for gender (appendix). Adjusting the treatment differences for gender did not change the conclusions, with large treatment effects still shown on all measures at all timepoints (data not shown).

No adverse events were reported by any participant in either treatment group. Levels of discomfort (as assessed by the SSQ) were very low before entering VR for the first time (n=47; mean total score 1·60 [SD 1·88]) and discomfort increased only slightly after being in VR (n=47; mean total score 3·81 [SD 3·80]; mean increase 2·21, 95% CI 1·24–3·18; p<0·0001). Before the last session of VR, levels of discomfort were also very low (n=37; mean total score 1·21 [SD 2·27]) and, again, increased only slightly after being in VR (n=37; mean total score 2·57 [SD 3·98]; mean increase 1·35, 95% CI 0·55–2·16; p=0·002).

## Discussion

The findings of our large randomised controlled trial show that an automated psychological intervention delivered by immersive VR is highly effective for reduction of fear of heights. All participants were followed up at every timepoint, meaning that the treatment effect estimates were unbiased by missing data. Treatment uptake was very high, indicating that the VR intervention was well received. Levels of discomfort after a VR session were very low, particularly since trial participants were facing their feared situation.

Findings of a meta-analysis indicated a mean effect size reduction in phobias with therapist-assisted exposure treatment (using real heights) of d=1·1;^19^ our automated VR treatment produced effect sizes that greatly exceeded this value (d=2·0). Therefore, the treatment effects produced were at least as good as—and most likely better—than the best psychological intervention delivered face-to-face with a therapist.

The initial cost of software development was reasonably high, with a team comprising psychologists, programmers, script writers, and an actor working intensively over approximately 6 months, but subsequent costs for this treatment are very low, with no need for a therapist to be present and inexpensive consumer VR hardware used. As VR becomes more widespread in households, such a treatment could be used at home in the future. Our view is that automated immersive VR has the potential to increase access to the best psychological interventions radically.

Our trial has several limitations. First, we do not know how representative our participants are of the wider population of people with acrophobia. Second, we relied on established acrophobia questionnaires and did not test behaviour at real heights. Third, we did not test long-term outcomes of the VR treatment, because previous studies of VR-assisted therapy have shown that reductions in anxiety can last several years.^20^, ^21^ Fourth, since most people do not receive treatment for a fear of heights, the clinical question addressed in the trial was the pragmatic one of how effective in total was the VR treatment against the absence of other treatment. Therefore, we can only conclude that the automated VR treatment produces large reductions in fear of heights, but we cannot pinpoint which elements caused the reduction. For example, we do not know whether similarly clinically effective automation of treatment can occur without use of a virtual coach. Fifth, the VR treatment was brief, and it is possible that further benefits might occur with a longer duration of treatment. Increased effectiveness might also occur with integration of the VR treatment into behavioural experiments in the real world. We are piloting provision of the VR treatment over half a day, and the outcomes are similarly encouraging. Finally, the extent to which learning from our work will transfer to other mental health conditions, particularly those seen in secondary care services, is uncertain. Automated treatment of anxiety disorders using VR might be a more tractable problem than for other disorders. We believe that transferability can only be determined by investment in high-quality VR treatments that are then tested in clinical trials. This endeavour is very important in mental health research in view of the potential benefits that might result from greatly increasing access to evidence-based treatment for mental health disorders.
