# Supplementary material for: Automated psychological therapy using immersive virtual reality for treatment of fear of heights: a single-blind, parallel-group, randomised controlled trial
Source: Lancet Psychiatry. 2018 Aug;5(8):625–32. doi: 10.1016/S2215-0366(18)30226-8 (PMC6063994; doi:10.1016/S2215-0366(18)30226-8)
Supplement: Supplementary appendix [file mmc1.pdf]

THE LANCET
Psychiatry

## Supplementary appendix

This appendix formed part of the original submission and has been peer reviewed. We post it as supplied by the authors.

Supplement to: Freeman D, Haselton P, Freeman J, et al. Automated psychological therapy using immersive virtual reality for treatment of fear of heights: a single-blind, parallel-group, randomised controlled trial. *Lancet Psychiatry* 2018; published online July 11, 2018. http://dx.doi.org/10.1016/S2215-0366(18)30226-8.

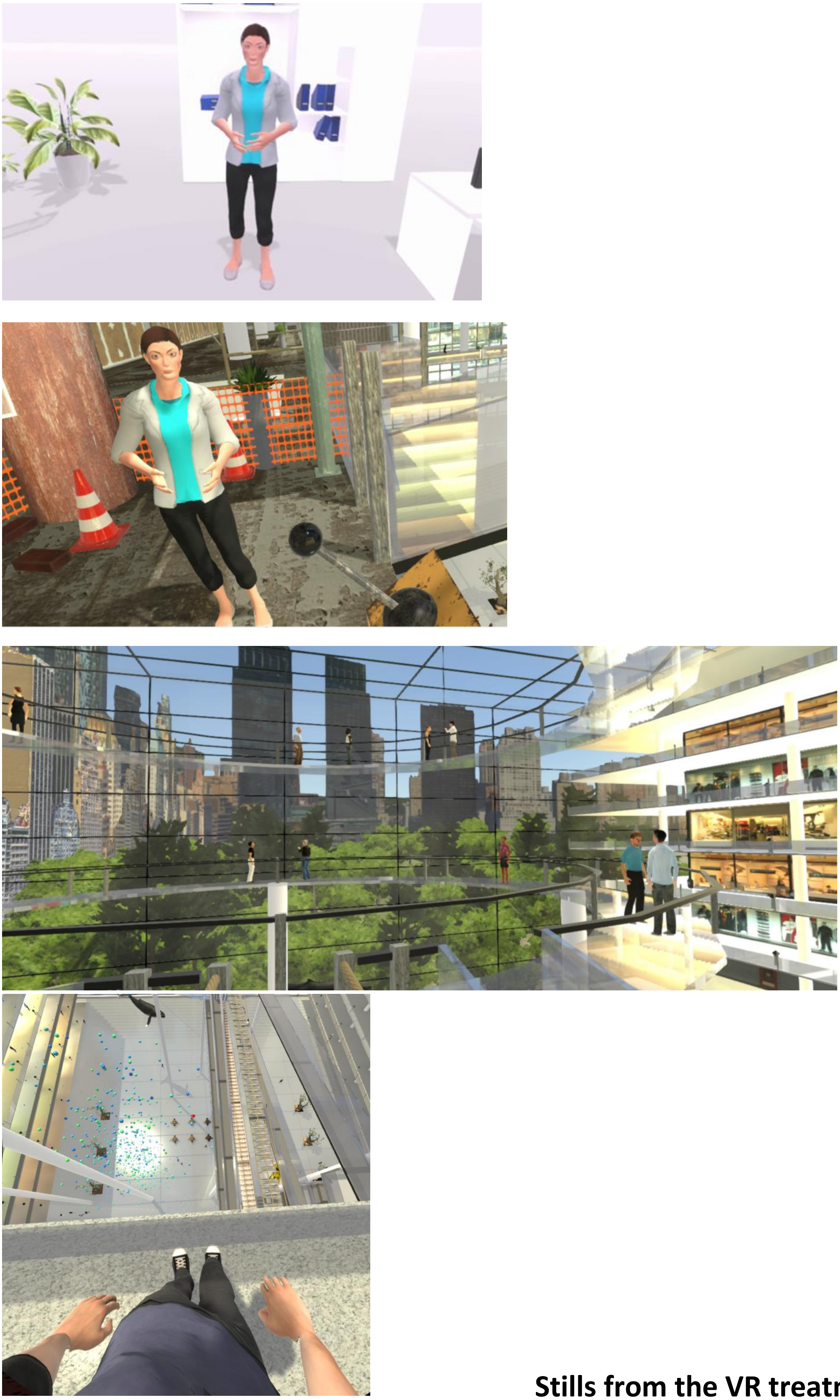

**Stills from the VR treatment**
